# ASC nanobodies to counteract the consequences of inflammasome activation

**DOI:** 10.15252/emmm.202216087

**Published:** 2022-05-16

**Authors:** Sahil Adriouch, Pablo Pelegrin

**Affiliations:** ^1^ UNIROUEN INSERM U1234 (PANTHER) Normandie University Rouen France; ^2^ Biomedical Research Institute of Murcia (IMIB) University of Murcia Murcia Spain

**Keywords:** Immunology, Musculoskeletal System

## Abstract

Inflammasomes are multiprotein complexes that signal by oligomerizing the apoptosis speck‐like protein with caspase recruitment and activator domain (ASC) and are involved in multiple inflammatory, metabolic and degenerative diseases. Pharmacological targeting of specific inflammasomes with small molecules is leading to the development of novel drugs for most common diseases. The targeting of ASC oligomers will result in a pan‐inflammasome treatment. In their study, Bertheloot *et al* (2022) developed specific anti‐ASC nanobodies and showed their efficacy to disaggregate already formed ASC oligomers and to treat inflammatory diseases in animal models. This approach represents a novel biologic‐based treatment for inflammasomes‐initiated inflammatory diseases.

## Graphical abstract

P. Pelegrin & A. Sahil discuss the study by Bertheloot et al (in this issue of EMBO Mol Med) that reports specific anti‐ASC nanobodies able to disaggregate ASC oligomers and treat inflammatory diseases in animal models.

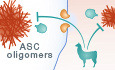

Inflammasomes are composed of a sensor protein that, upon activation, recruit ASC into large helicoidal filaments that activate caspase‐1 by bringing pro‐caspase‐1 zymogens into close proximity. Caspase‐1 is the effector enzyme of the inflammasome, and by cleaving the pore‐forming protein gasdermin D (GSDMD), it mediates a specific type of necrotic‐like cell death called pyroptosis. The resulting N‐terminal fragment of GSDMD inserts into the plasma membrane and self‐oligomerizes, leading to the formation of pores of around 10–15 nm in inner diameter that allows the release of pro‐inflammatory cytokines and other intracellular content able to initiate and amplify the inflammatory response (Broz *et al*, 2020). GSDMD pore leads to consequent permeabilization of the plasma membrane and the release of large‐cellular associated content, as ASC oligomers and other pro‐inflammatory intracellular mediators that further propagate inflammasome signalling (Baroja‐Mazo *et al*, 2014; Franklin *et al*, 2014). In fact, extracellular ASC oligomers have been implicated in the pathophysiology of multiple inflammatory‐related diseases, such as chronic obstructive pulmonary disease, cryopyrin‐associated periodic syndromes, sepsis, Alzheimer’s disease, non‐alcoholic fatty liver disease and liver fibrosis (Baroja‐Mazo *et al*, 2014; Franklin *et al*, 2014; Venegas *et al*, 2017; Martínez‐García *et al*, 2019; Gaul *et al*, 2020). Therefore, pharmacological targeting of inflammasomes has been an area of intense research and investments by small biotech and big pharma companies. This has led to the development of small molecules targeting specific inflammasome sensor proteins, to avoid their activation (Coll *et al*, 2022). However, this might lead to (i) restrict their use to the diseases where the specifically targeted inflammasome sensor is involved, and (ii) reduce their therapeutic effects in already established inflammatory diseases, where post‐inflammasome actors perpetuate the signaling by releasing the pro‐inflammatory content of the cells dying by pyroptosis, which include the release of non‐reversible prion‐like ASC oligomers. Therefore, pyroptosis consequences would be hardly blocked by such inflammasome inhibitors, and novel blockers of GSDMD‐induced pyroptosis are being developed (Coll *et al*, 2022). However, while blocking pyroptosis might appear as a novel approach to inhibit post‐inflammasome‐related signaling, inflammasome activation and accumulation of active caspase‐1 may continue, potentially leading to alternative GSDMD‐independent cell death and to the release of their pro‐inflammatory content. In this context, targeting ASC is emerging as an alternative pathway to block multiple inflammasomes, and the small molecule MM01 has been recently identified as a blocker of ASC oligomerization triggered by different inflammasome sensors therefore, able to mitigate the disease in a pre‐clinical model of gout (Soriano‐Teruel *et al*, 2021). Similarly, the use of anti‐ASC antibody to therapeutically neutralize ASC after its release from dying cells has reduced inflammasome signaling and its consequences in a rat model of traumatic brain injury (de Rivero Vaccari *et al*, 2009). In this issue of EMBO Molecular Medicine, Bertheloot *et al*. identify a novel ASC nanobody. Nanobodies, represent a promising novel class of biologic derived from single chain only camelid‐antibodies. The antigen‐binding domain derived from those camelid‐antibodies can hence be used alone to produce a single‐domain binding module, named VHH or nanobody, which represents the smallest known type of antibody. Here, the authors have generated and selected an anti‐ASC nanobody based on its ability to bind and inhibit ASC oligomerization. Interestingly, the selected nanobody was also able to disaggregate already formed ASC oligomers inside the cells, provided that the nanobody gain access to the intracellular compartment, as well as ASC oligomers present in the extracellular milieu (Bertheloot *et al*, 2022). The authors demonstrated that the nanobody could indeed enter the cell through the GSDMD‐pore induced at a late stage by inflammasome activation, resulting in the earlier termination of inflammasome activation and subsequent consequences (Fig 1). Contrary to classical antibodies, the small size of nanobodies, around 4 nm long and 2.5 nm wide, makes them compatible with entry through GSDMD‐pores (estimated size of 10–15 nm). As this mechanism does not prevent early inflammasome activation, it is anticipated that such a strategy might not completely inhibit all the major infectious defence mechanisms, as opposed to therapeutic strategies that completely inhibit innate immunity, thus increasing the risk of infections. Perhaps more importantly, the selected nanobody was able to target extracellular ASC oligomers and prevent their extracellular signalling effects that are known to perpetuate and amplify inflammation in the vicinity of the cell dying from pyroptosis. Moreover, the nanobody was also demonstrated to disrupt the “prionoid” activities of ASC oligomers in the extracellular milieu that are supposed to promote the oligomerization of soluble ASC and to further aggravate inflammation (Fig 1). Finally, the authors demonstrated the therapeutic efficacy of their anti‐ASC nanobody in a pre‐clinical model of gout and in a model of antigen‐induced arthritis (Bertheloot *et al*, 2022). This study broadens the toolbox to more precisely decipher the consequences of post‐inflammasome signalling in the perpetuation of inflammation and tissue injury. This can be achieved in acute inflammatory models using direct anti‐ASC nanobody injections, as well as in chronic disease, using for instance viral gene therapy vector to promote long‐term expression in vivo. Such approaches could also be used to evaluate the possible relation between ASC‐prionoid activity and amyloid aggregates in the brain using models of Alzheimer’s disease. Therefore, anti‐ASC blocking nanobodies represent the next‐generation of inflammasome‐targeting biologics to tackle the detrimental consequences of inflammasome downstream signalling in already established inflammatory diseases.

**Figure 1:**
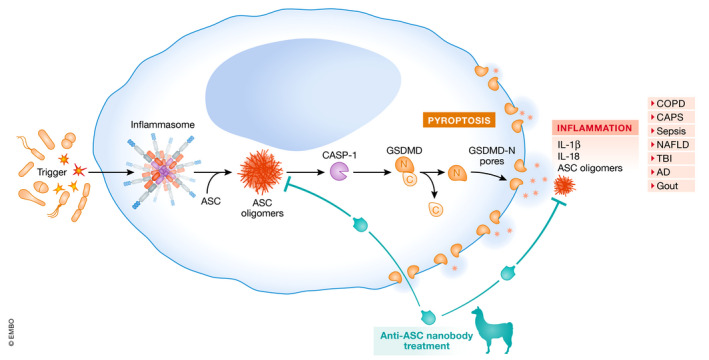
Mechanism of action of anti‐ASC nanobody targeting ASC specks The activation of the inflammasome result in a large oligomer of ASC that is released during pyroptosis, leading to extracellular inflammatory functions in different diseases. ASC targeting nanobodies have the potential to disassemble ASC oligomers extracellularly and intracellularly, by penetrating the cell through gasdermin D pores. AD, Alzheimer's disease; CAPS, Cryopyrin‐associated periodic syndrome; COPD, Chronic obstructive pulmonary disease; NAFLD, Nonalcoholic fatty liver disease; TBI, Traumatic brain injury.

## Disclosure statement and competing interests

PP is the founder and consultant of Viva in vitro diagnostics SL, a company dedicated to use inflammasome for clinical diagnostics, and is also a consultant of Glenmark Pharmaceuticals and Monte Rosa Tex companies interested to develop inflammasome blockers. SA declare no conflict of interest.
